# New insights in otology and neuro-otology^☆^

**DOI:** 10.1016/j.bjorl.2015.08.003

**Published:** 2015-09-08

**Authors:** Carlos Augusto Costa Pires de Oliveira

**Affiliations:** Programa de Pós-Graduação, Faculdade de Medicina, Universidade de Brasília (UnB), Brasília, DF, Brazil

Research and innovation are certainly the drivers behind the progress of medicine. However, physicians must be careful in electing changes to implement. Things are not always beneficial simply because they are new. Old concepts may stand up to time and become classics. In Brazil, most of the time we tend to accept and start incorporating new ideas and concepts from abroad. Worse, we tend to discard old concepts as soon as we come across new ones. Frequently, old ideas are still valid and can help us together with the new ones; thus, it is wise to select new ideas while keeping the time-tested ones alive.

Subspecialization is certainly a concept that has contributed greatly to Otolaryngology. However, it must be built upon a solid general basis. The general understanding of head and neck medicine and surgery will make possible a permanent dialog among specialists. It is the common ground that will keep Otolaryngology professionals together and communicating with each other. Isolated micro-subspecialties will never be strong enough.

It follows that our residency programs must be able to teach all aspects of head and neck medicine and surgery. A good program must not allow residents to become subspecialized during their training; excellence in some fields is not an acceptable substitute for good teaching in all aspects of head and neck care.

The excellence of a residency program should be measured by its ability to teach the broad capacities of an otolaryngologist-head and neck surgeon, just as the ABORL description of an otolaryngologist's capabilities states.

This is the way it works in the United States, and certainly this philosophy has made Otolaryngology a long-standing, strong, and respected specialty. Are we following these guidelines in Brazil?

How are we to produce the specialists in the subareas of our field? Some decades ago it was possible to teach Otolaryngology-Head and Neck Surgery in a four-year residency program. This is no longer possible because of the enormous progress that has taken place in head and neck medicine and surgery. The Residency must produce general otolaryngologists with basic capabilities in all fields of our specialty. Subspecialization must be pursued in fellowships to be embarked upon after the residency. Excellent residency programs should be able to teach the residents all aspects of otolaryngology as well as teach fellows in certain subspecialized areas.

What does all this jazz has to do with new insights in Otology and Neuro-Otology?

The international Neurootology and Equilibriometric Society (NES) was set up by medical doctors, technical assistants, and engineers interested in neuro-otology during a meeting in the Head and Neck Clinic of the Neuro-otology Division of the University of Wursburg on May 25, 1974. Gottfried Aust, Christian Betow, and Erika Claussen from Berlin; Claus-Frenz Claussen from Wursburg; Christian Henning from Ulm; Gunter Hortman (electrical engineer); and Pedro Estelrrich and Juan Manuel Tato from Argentina are among the founders.

At that initial meeting, it was established that the NES would be concerned with the diagnosis of neuro-otological malfunction or dysfunction, comprising the fields of the human “Exosensorium”: equilibriometry (vertigo, dizziness, etc.), audiometry (hearing disorders, tinnitus, etc.), olfactometry (dysosmia, anosmia, etc.) and gustometry (dysgesia, ageusia).^1^

Since that time (1974), the NES has held annual meetings that have alternated between Bad Kissingen, Germany, and somewhere abroad the following year. Forty-one meetings were held from 1974 to 2014, and all continents have hosted at least one of them. The format of the meetings has been always the same: there are two sections in every Congress. One deals with neuro-otology and the other with tinnitus. Tinnitus has become an important part of the NES after Professor Abraham Shulman from New York State University joined the Society. Shulman has dedicated his life to the study of tinnitus and has produced quite an admirable body of concepts and theories about this symptom, which now are accepted as important contributions. The meetings are filled by scientific contributions from the members of the NES in the format of 15-min presentations that are discussed during each section of paper presentation.

The NES has embraced important contributions from Brazilian neuro-otologists during these years. Professors Yotaka Fukuda, Maurício, and Fernando Ganança of UNIFESP, and Professors Maudonet and Mezzalira from Unicamp have all contributed to the Society; one of the past NES meetings took place in São Paulo under the Chairmanship of Professor Maurício Ganança (1987).

Because of my association with Professor Shulman, I became involved with the NES to study tinnitus. In the last 18 years, I have attended several NES meetings and in 1914 in Prague, I was elected Vice President of the Society and received the mission of organizing the Congress in Brazil in 2015.

The idea was to organize the meeting in Brasília the same way it had been conducted in the past. However, it became clear to me that a meeting dealing exclusively with neuro-otology held in Brasília would be very difficult to carry out successfully. Also, the format of the NES meetings was certainly not compatible with the Brazilian concept of a congress.

When one is in trouble, help must be sought. So I asked Professor Fernando Ganança to participate in the organization of the meeting. He accepted, and indeed was with me all the time until we finished. We asked Stela Maris Events to organize the Congress. At this point, a fortunate thing happened: Professors Sady Selaimen and Oswaldo Laércio Cruz were organizing the first Ear Brazil to take place about the same time the NES Congress was scheduled to happen. Fernando then had the thought: why don’t we combine Ear Brazil and the NES Congress into one major event? I agreed immediately, and fortunately Sady and Laércio also promptly agreed.

We did not realize that this was a major innovation in the NES meetings. Soon we started to get feedback from the NES membership manifesting absolute disagreement with our idea of combining their meeting with another event, making the NES a partner in the scene.

We took a firm stand and decided to go ahead with our plan. We scheduled talks by Brazilian professors, we put together round tables mixing up otoneurologists and otologists discussing common themes, we maintained the sections for presentations of papers, and we hoped for the best.

The meeting was a success. We have received congratulations from Professor Claussen, Professor Szirmai, and members of the NES.

Indeed, the meeting had a very high scientific level thanks to the excellent papers presented by Brazilian graduate students and residents. Innovation here was clearly present, and blending otolaryngologists with different subspecialists to discuss common problems was a step toward maintaining a common basis for our specialty. The third day mixed up Ear Brazil and the NES Congress completely in a common program. Indeed, the final round table combining neuro-otologists and otologic surgeons was enlightening.

New insights in otology and neuro-otology certainly were born during this memorable experience. A well-balanced mix of innovation and good, solid tradition is certainly the way forward. Our past is solid. It certainly will enlighten our path to the future.

## Conflicts of interest

The author declares no conflicts of interest.

## References

[1] (2012). Annals of the XXXIX NES Congress.

